# Supplementary material for: Cancer patient survival can be parametrized to improve trial precision and reveal time-dependent therapeutic effects
Source: Nat Commun. 2022 Feb 15;13:873. doi: 10.1038/s41467-022-28410-9 (PMC8847344; doi:10.1038/s41467-022-28410-9)
Supplement: Supplementary file 5 — Supplementary Dataset 2 [file 41467_2022_28410_MOESM5_ESM.zip › Supplementary Data File 2/Weibull fits R2- Fig 2, S7/OS linearity.pdf]

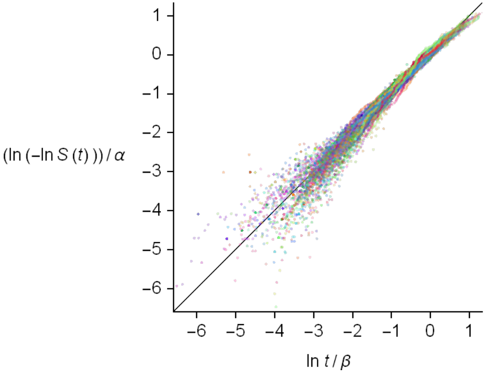

(ln (−ln S (t))) / α
ln t / β
1
0
−1
−2
−3
−4
−5
−6
−6
−5
−4
−3
−2
−1
0
1
